# A Dietary and Lifestyle Intervention Improves Treatment Adherence and Clinical Outcomes in Overweight and Obese Patients with Obstructive Sleep Apnea: A Randomized, Controlled Trial

**DOI:** 10.3390/life13081755

**Published:** 2023-08-16

**Authors:** Izolde Bouloukaki, Eleni Daskalaki, Eleni Mavroudi, Violeta Moniaki, Sophia E. Schiza, Ioanna Tsiligianni

**Affiliations:** 1 Department of Social Medicine, Faculty of Medicine, University of Crete, 71500 Heraklion, Greece; elenianastasiadaskalaki@gmail.com (E.D.); pdkapa@yahoo.gr (I.T.); 2 Sleep Disorders Center, Department of Respiratory Medicine, Medical School, University of Crete, 71500 Heraklion, Greece; elenima23@hotmail.com (E.M.); vmoniaki@yahoo.gr (V.M.); schizas@uoc.gr (S.E.S.)

**Keywords:** dietary intervention, obstructive sleep apnea, treatment adherence, sleepiness, obesity, Mediterranean diet

## Abstract

The study’s objective was to assess the impact of Mediterranean diet/lifestyle interventions for weight loss on positive airway pressure (PAP) adherence, body mass index (ΒΜΙ), sleepiness, and blood pressure measurements (BP) in patients with obstructive sleep apnea (OSA). We designed a randomized, controlled trial, including overweight and obese patients with moderate to severe OSA, randomized to standard care (SCG, n = 37) or a Mediterranean diet group (MDG, n = 37). The SCG received healthy lifestyle advice, while the MDG underwent a 6-month behavioral intervention aiming to enhance weight loss and adherence to a Mediterranean diet. PAP adherence, BMI, Epworth Sleepiness Scale (ESS), and BP measurements were evaluated pre- and post-intervention. Post-intervention PAP use was higher in the MDG compared to the SCG (6.1 vs. 5.4, *p* = 0.02). Diet/lifestyle intervention was one of the most significant predictive factors for PAP adherence (OR = 5.458, 95% CI = 1.144–26.036, *p* = 0.03). The SCG demonstrated a rise in BMI, while the MDG displayed a decline (0.41 vs. −0.75, *p* = 0.02). The MDG also demonstrated a substantial reduction in adjusted SBP (−5.5 vs. 2.8, *p* = 0.014) and DBP (−4.0 vs. 2.5, *p* = 0.01). Ultimately, incorporating a dietary/lifestyle intervention with standard care yields superior PAP adherence, BMI, and BP measurements in contrast to standard care alone, emphasizing the advantages of dedicating more time and support within the MDG.

## 1. Introduction

Obstructive sleep apnea (OSA) is a common and under-recognized public health problem, associated with increased cardiovascular morbidity and mortality and significant increases in health and social costs [1,2,3,4]. Obesity has long been acknowledged as one of the most significant risk factors for OSA [5]. Furthermore, the prevalence of OSA increases with adiposity and ranges between 50 and 80% for individuals who are classified as overweight or obese [6].

OSA should be approached as a chronic disease that requires pathophysiological and clinical phenotyping, objective diagnostic testing and individualized treatment plans with positive airway pressure (PAP) as the first-line symptomatic treatment of choice. PAP treatment in adherent patients with OSA yields several benefits, such as improved daytime sleepiness, systemic blood pressure, quality of life, neurobehavioral performance, and a decreased risk of motor vehicle accidents [7,8,9]. However, PAP acceptance and compliance remain a challenging issue [10]. Therefore, it is crucial to implement strategies for improving and sustaining adherence over time in these patients. These strategies should go beyond the traditional mask adjustment and leak assessment, and further integrate the involvement of multidisciplinary teams [11].

Given the strong association between obesity and OSA, lifestyle interventions have emerged as complementary therapeutic choices. In line with this, the American Heart Association recommends including weight-loss-focused lifestyle interventions alongside conventional OSA treatment [2]. Lifestyle-induced weight loss has been extensively investigated as a treatment approach to reverse OSA pathogenesis and is effective in improvement in both OSA severity and OSA-related symptoms [12,13,14]. A combination of a 6-month behavioral dietary/lifestyle modification program based on the Mediterranean pattern and PAP therapy was found to effectively reduce weight, improve OSA severity, and result in favorable anti-inflammatory, antioxidant, and cardiometabolic outcomes in OSA patients [15,16,17]. While weight regain is expected in the long-term, these cardiometabolic benefits appear to be sustainable even after six months following the intervention [18].

Besides improvements in OSA severity and cardiometabolic parameters, no evaluation has been undertaken to determine the effect of diet/lifestyle interventions on objective PAP adherence. Since adherence is one of the major determinants of PAP efficacy, we hypothesized that expanding the scope of diet/lifestyle interventions beyond OSA severity and cardiometabolic parameters could enhance PAP adherence among these patients. Therefore, the aim of our study was to explore the role of a 6-month diet/lifestyle intervention on treatment adherence and clinical outcomes in patients with OSA. Specifically, we evaluated the effects of a combination of PAP and weight-loss Mediterranean diet/lifestyle intervention on improving PAP adherence (hours of device use), body mass index (ΒΜΙ), and daytime symptoms, mainly sleepiness and arterial blood pressure measurements, over the effect of usual (standard) care alone.

## 2. Materials and Methods

### 2.1. Study Patients

We conducted a parallel, randomized, controlled, follow-up study of consecutive patients who were admitted to the Sleep Disorders Center, Department of Respiratory Medicine, University of Crete Medical School, between December 2021 and March 2022. The inclusion criteria were (a) patients aged >18 years with newly diagnosed moderate to severe OSA (apnea-hypopnea index (AHI) ≥ 15 events/h) through an attended overnight polysomnography according to standard criteria, (b) overweight and obese (BMI > 25 kg/m^2^), (c) eligible for PAP treatment with a follow-up in-laboratory PAP titration with full polysomnography to establish the appropriate PAP settings, (d) with adherence data available in the 6 months after initiation of treatment, and (e) with an above-elementary school education. The exclusion criteria were refusal to participate, patients on PAP treatment, current participation in a weight loss program, central sleep apnea syndromes, obesity hypoventilation syndrome, restrictive ventilator syndromes, severe congestive heart failure, a history of life-threatening arrhythmias, severe cardiomyopathy, long-term oxygen therapy, chronic kidney disease, family or personal history of mental illness, drug or alcohol abuse, severe cognitive impairment, concurrent oncological diseases, pregnancy or lactation, recent hospitalization for acute or chronic respiratory disease, history of narcolepsy, or restless leg syndrome. All subjects provided written informed consent and ethical approval was provided by the University of Crete Research Ethics Committee (REC-UOC) (approval number: 158/29.11.2021). The trial was also registered on ClinicalTrials.gov with Trial registry: ClinicalTrials.gov; No.: NCT05881824. 

**Figure 1:**
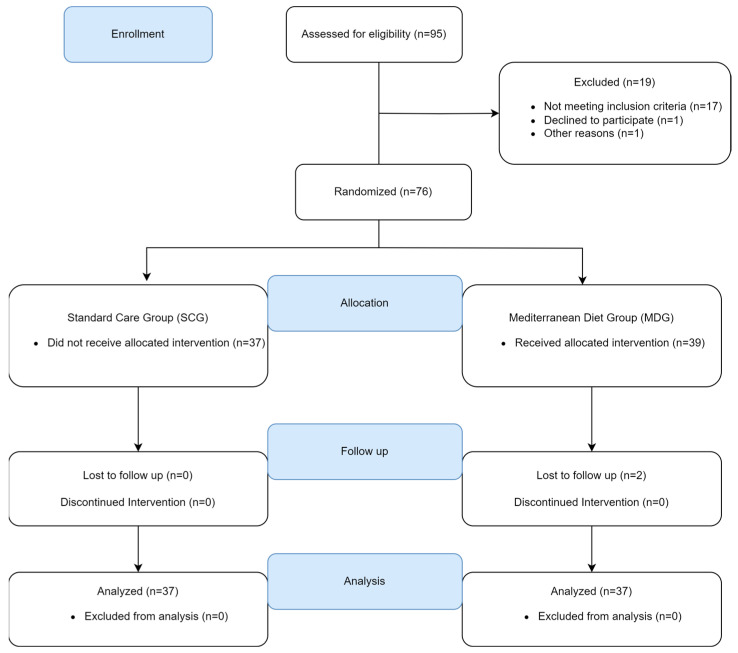
Flowchart of the study.

Individuals were assigned (1:1) following simple randomization procedures (computerized random numbers) to a usual (standard) care group (SCG, n = 39) receiving usual follow-up care or an intervention group—Mediterranean diet group (MDG, n = 37) with follow-up care based on an additional behavioral intervention aiming at weight loss and increasing adherence to the Mediterranean diet (Figure 1). Following randomization, sequentially numbered envelopes that were opaque and sealed were utilized in the allocation concealment process, prepared by an individual not involved in the trial.

Blinding the patients or care team was not feasible since all patients were informed in the consent form that they would be randomized to either the MDG or SCG. Study investigators and the statistician involved in data analysis were blinded to the intervention.

Patients were followed for 6 months.

### 2.2. Data Collection

All patients underwent a detailed evaluation that included anthropometric parameters including BMI, and medical and sleep history, and comorbidities including physician-based diagnosis for depression, smoking history, and alcohol intake. Subjective daytime sleepiness, reflected by the Epworth Sleepiness Scale (ESS), and the patient’s level of depression reflected by Beck’s depression scale (BDI) were also recorded before PAP initiation. ESS, PAP adherence (hours of device use), BMI, and arterial blood pressure measurements were also evaluated pre- and post-intervention.

#### 2.2.1. Epworth Sleepiness Scale (ESS)

The ESS is currently the most widely used subjective test of daytime sleepiness in clinical practice [19]. A score of 10 or higher represents excessive daytime sleepiness.

#### 2.2.2. Beck Depression Inventory (BDI)

This 21-item questionnaire is a widely used and well-validated self-reported inventory of depressive symptoms [20]. The BDI measures the severity of depressive symptoms over the preceding week. For each item, the respondent chooses one or more options rated from 0 (absence of symptoms) to 3 (most severe level). Total scores range from 0 to 63 and represent the sum of the highest level endorsed on each item. Scores below 10 are considered normal.

### 2.3. Follow-Up—Usual (Standard) Care Group

All patients attended a PAP clinic before treatment initiation, where they were given specific counseling and education on the proper use and maintenance of PAP and underwent personalized, formal mask fitting by a specialized nurse. The total time for the appointment in the PAP clinic was 20 min/patient. Once PAP was started, patients were reviewed in the outpatient sleep clinic at 1-month and at 3-month intervals during the first year, and every 6 months thereafter. During these appointments, a clinical assessment was made, and patients were further encouraged to use the device. In addition, all patients received oral healthy lifestyle advice and counseling on physical activity and sleep habits and had the opportunity to discuss other health issues related to the condition, such as weight reduction and smoking cessation. At each visit, the compliance data were downloaded from the PAP device and reviewed by the PAP clinic nurse together with the patients. Any concerns or questions, such as pressure sores, persistent air leakage, claustrophobia, nasal congestion, and other side effects resulting from the nasal mask interface that might lead to suboptimal compliance were addressed immediately by the PAP clinic nurse. Changes in the PAP setting, nose/face mask, or circuit were made after consultation with the responsible sleep physician if necessary. In every follow-up visit, residual symptoms, including residual sleepiness, or change in patient’s overall health status were recorded by the sleep nurse and sleep physician. This format adhered to a standardized approach according to our PAP clinic’s procedures [21].

### 2.4. Follow-Up—Intervention Group

All patients in the intervention group attended individual weekly 60–90 min sessions led by a dietitian in the first month and twice/month thereafter. In this group, all the features described above for the standard group were included, plus additional visits involving intensive dietitian-led behavioral intervention aiming at weight loss and increasing adherence to the Mediterranean diet [22]. Dietary behavior was assessed through the Food Frequency Questionnaire [23] and Mediterranean Diet Score [24] before PAP initiation. The dietitian conducted a 24 h recall for the participant, aiming to gather data on his customary dietary program, meal timings, preferred food quantities, and quality. At this point, questions were also posed regarding the consumption of water, alcohol, smoking, any allergies, special preferences or aversions, and about physical activity. In addition, a comprehensive approach was adopted by obtaining data on family, cultural, and professional background in order to guide subsequent steps towards the maximum possible outcome.

Subsequently, the dietitian provided every participant in this group with a document containing comprehensive suggestions for a nutritious diet, illustrating all food categories and outlining the specific amounts for each, clarifying which options are healthier for each group (e.g., lean dairy). The dietitian engaged in a dialogue with the participant to gather further details or inquiries about the diet, following which the former devised a personalized diet plan based on the latter’s requirements, inclinations, and unique characteristics.

Accordingly, this intervention was adjusted to fulfill the specific needs of these patients. Guidance in physical exercise, optimal sleep length, and sleep hygiene education were also given. Ultimately, a personalized therapeutic diet plan was implemented in this group.

#### 2.4.1. Food Frequency Questionnaire

The FFQ used in this study has previously been demonstrated to be reproducible and relatively valid to assess practically all food groups, as well as macronutrients and energy consumption [23]. It includes 75 items (foods and beverages commonly consumed in Greece and dietary habits). The amounts of food consumed were expressed in grams or milliliters or in other common measures, such as slice, tablespoon or cup, representing the standard serving size. On a 6-point scale, participants were asked to report how frequently they consumed each of the meals and beverages listed in the FFQ on average over the period of one month preceding the study period (never/rarely, 1–3 times/month, 1–2 times/week, 3–6 times/week, 1 time/day, or ≥2 times/day).

#### 2.4.2. Mediterranean Diet Score

The Mediterranean Diet Score (MedDietScore) is a 14-item validated questionnaire produced for each participant to assess their level of adherence to the Mediterranean Diet, taking into consideration their consumption of food items from nine food groups, as well as olive oil and alcoholic beverages [24]. Each of the 14 items is scored one or zero, depending on whether participants adhere to each MedDiet component or not. The Mediterranean Diet Score has a range of 0–55, with higher values indicating greater adherence to the Mediterranean Diet.

### 2.5. PAP Adherence

PAP usage data included mask type (nasal or full face), number of nights on PAP, average use per night (hours), air leakage, and air pressure delivered. The usage of PAP, effective pressure, and residual AHI was monitored at 1, 6, 12 months after initiation, and patients were contacted by a trained sleep nurse. Patients were also encouraged to contact the telephone helpline during working hours. The humidification was defined based on patients’ feedback and was adjusted during the follow-up if necessary. In order to optimize PAP adherence unplanned visits were immediately arranged in case of low adherence to PAP therapy. Regular PAP compliance was defined as using the therapy for an average of 4 h a night for at least 70% of the nights [25]. However, in our study for optimal PAP adherence we used the cut-off point of six hours of PAP use [21,26].

### 2.6. Statistical Analysis

A pilot study was conducted with 49 individuals to determine the sample size. With the data obtained from the pilot study, the sample size was determined as at least 63 individuals, to obtain at least 80% power to detect a significant difference in the follow-up mean hours of PAP use values between the MDG and the SCG, allowing for a type-I error rate of 0.05.

Results are presented as mean ± standard deviation (SD) for continuous variables if normally distributed and as median (25th–75th percentile) if not. Qualitative variables are presented as absolute number (percentage). For comparisons between groups, a two-tailed t-test for independent samples (for normally distributed data) or a Mann–Whitney U test (for non-normally distributed data) was utilized for continuous variables and the chi-square test for categorical variables. The analysis of covariance was used to test adjusted between-group differences at the end of the 6-month intervention. All models were adjusted for basic confounders, namely age and gender, baseline BMI, smoking status, questionnaires scores, OSA severity indices, and co-morbidities. Analyses were additionally adjusted for weight loss (expressed as BMI difference) to test the weight-loss independent impact of the dietary/lifestyle intervention implemented on PAP adherence. Factors associated with optimal adherence (use of the device ≥6 h) at the end of the 6 months’ follow-up were analyzed with bivariate logistic regression after adjustment for various potential basic explanatory confounders. We checked multicollinearity among the predictors using collinearity statistics to ensure that collinearity between predictor variables was in the acceptable range as indicated by the tolerance value variance inflation factor. Age was considered continuously and categorically, as age groups of 18–59 and >60 years, BMI was also considered continuously and categorically, as BMI groups of <30 and ≥30 kg/m^2^. For the purpose of this analysis, the term cardiovascular disease (CVD) used, referred to any of the following conditions: coronary disease, stroke, atrial fibrillation, and heart failure. Results were considered significant when *p* values were <0.05. Data were analyzed using SPSS software (version 25, SPSS Inc., Chicago, IL, USA).

## 3. Results

Of 96 individuals with suspected OSA screened, 18 were non-eligible (mild OSA, normal weight, presence of other chronic diseases, etc.), one declined participation, and the remaining 76 were enrolled and randomized (SCG: 37, MDG: 39). After enrollment, 2 participants were excluded (lost to follow-up), leaving a final sample of 74 patients for analysis (SCG: 37, MDG: 37).

### 3.1. Comparison of Baseline Characteristics between the SCG and the MDG 

Participants’ socio-demographic and health status characteristics are outlined in Table 1. Most of the participants were men (78%), obese (77%), and current or former smokers (64%) with a medium educational level. The most prevalent diseases were hypertension (49%), followed by dyslipidemia (39%), CVD (27%), and COPD (22%). There was a significantly higher proportion of men in the intervention group compared to the control group. Other evaluated features remained relatively insignificant between both groups, such as age, other comorbidities, and smoking status (all *p* > 0.05), except for the presence of cardiovascular disease (CVD; 41 vs. 14%, *p* = 0.009).

Comparison of PSG parameters of the SCG and the MDG population showed no significant differences between groups (Table 2). Although no significant difference was noted between nocturnal and diurnal symptoms (Table 3), frequent awakenings reported were significantly higher in the MDG compared to the SCG (65 vs. 41%, *p* = 0.04).

Regarding lifestyle habits, the MDG exhibited a moderate level of adherence to the Mediterranean diet as assessed by MedDietScore (29 ± 5).

### 3.2. PAP Adherence

All patients continued to use their PAP at the end of the follow-up period. Auto-PAP was prescribed to the majority of participants with final levels at the end of the follow-up of PAP pressure of 9.2 for the SCG and 8.4 for the MDG (*p* = 0.04). Post-intervention PAP use was significantly higher in the MDG compared to the SCG (6.1 ± 1.2 vs. 5.4 ± 1.4, *p* = 0.02). Further analysis showed that this difference persisted after adjustments for age, gender, BMI, difference in BMI, ESS, difference in ESS, BDI score, OSA severity, and comorbidities (5.2 vs. 6.1, *p* = 0.03). Moreover, diet/lifestyle intervention was identified as one of the most significant predictive factors for optimal PAP adherence (OR = 5.458, 95% CI = 1.144–26.036, *p* = 0.03) (Table 4).

### 3.3. Effect of Diet Intervention on Anthropometric and Daytime Symptoms Parameters

**Figure 2:**
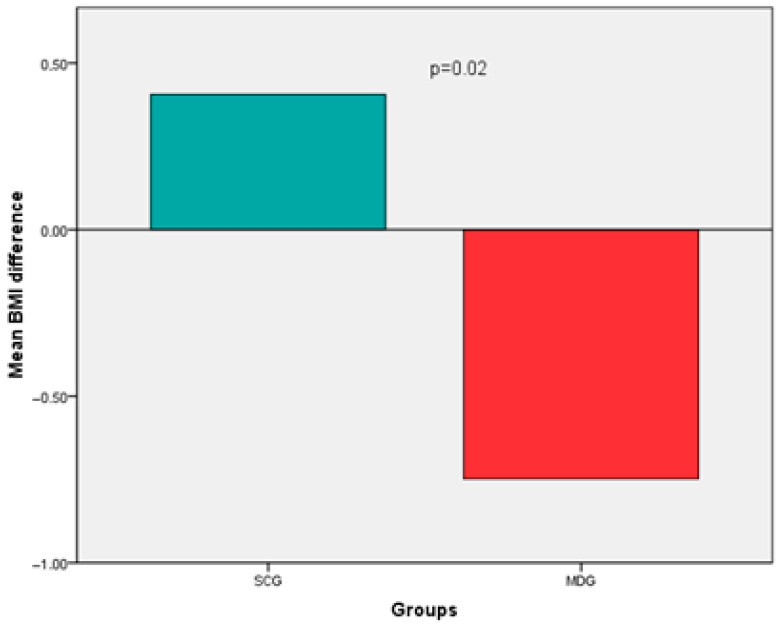
Post-treatment BMI differences between the SCG and the MDG.

Regarding BMI, an increase was noted in the SCG, whereas a decrease (improvement) was observed in the MDG, (0.41 ± 1.8 vs. −0.75 ± 1.3, *p* = 0.02) (Figure 2). Specifically, during the 6-month follow-up, patients in the SCG demonstrated an average weight gain of 1.6% of their baseline body weight, while those in the MDG showcased a loss equivalent to 1.5% (*p* = 0.04). BMI difference, although attenuated, persisted after adjustments for age, gender, BMI, ESS, BDI score, OSA severity, PAP adherence, and comorbidities (0.23 vs. −0.55, *p* = 0.31).

**Figure 3:**
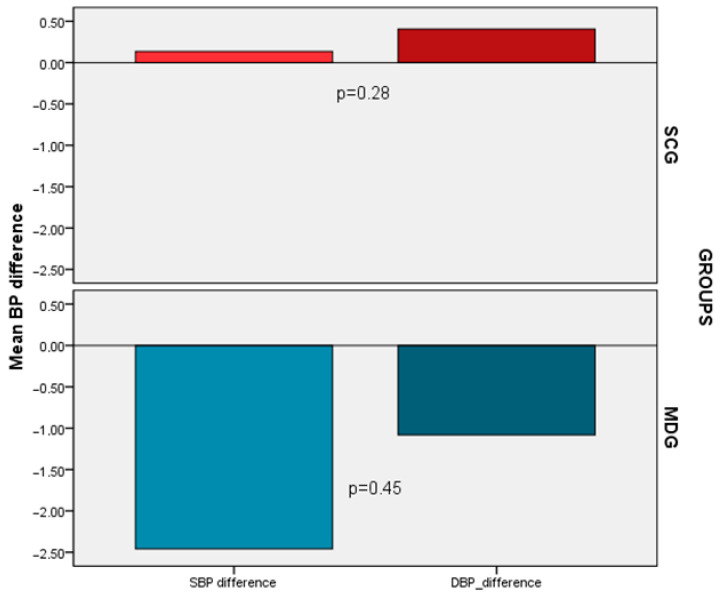
Post-treatment BP differences between the SCG and the MDG.

In terms of blood pressure measurements a decrease was noticed only in the MDG (SBP −2.5 ± 11, DBP −1.1 ± 7.6) compared to the SCG (SBP 0.14 ± 9.2, DBP 0.40 ± 9.2) after the 6 months of the follow-up period. Nevertheless, the aforementioned changes did not exhibit statistical significance (*p* = 0.28 and *p* = 0.45, respectively) (Figure 3). However, after accounting for confounding variables, a substantial decline in SBP (−5.5 vs. 2.8, *p* = 0.014) and DBP (−4.0 vs. 2.5, *p* = 0.01) was specifically detected in the MDG versus the SCG.

**Figure 4:**
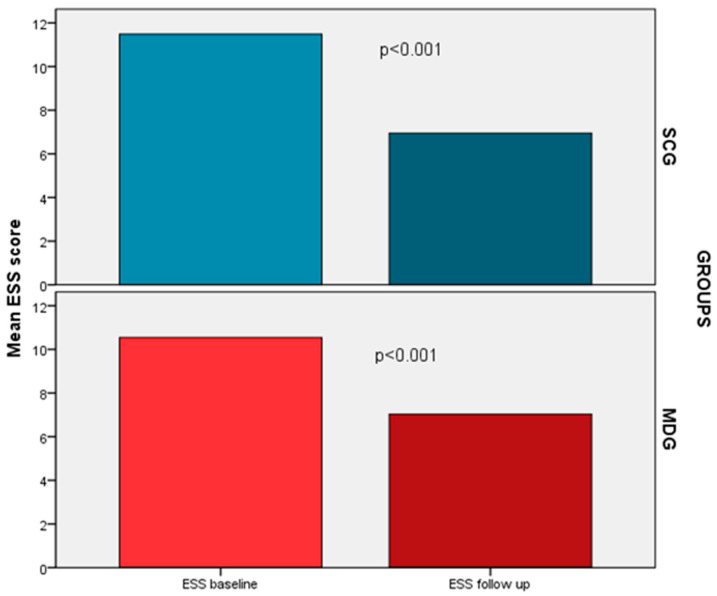
Post-treatment ESS score differences between the SCG and the MDG.

A significant decrease in ESS was also noted in both groups. Nonetheless, no group manifested a significant predominance in this improvement (−4.5 vs. −3.5, *p* = 0.19), even following adjustment of confounders (Figure 4). Additionally, both groups displayed a noteworthy decline in the proportion of patients experiencing excessive daytime sleepiness (SCG: 19% vs. 62%, *p* < 0.001, MDG: 16% vs. 54%, *p* < 0.001). The proportion of residual sleepiness at the end of the follow-up period was comparable between the groups (16% versus 19%, *p* = 0.76).

## 4. Discussion

Our study assessed the impact of dietary/lifestyle intervention along with usual care on PAP adherence, BMI, BP measurements, and sleepiness in moderate to severe OSA patients. The intervention demonstrated significant and clinically meaningful improvements in PAP adherence, BMI, and BP measurements in the intervention group compared to the control group, independent of age, gender, BMI, weight loss, baseline level of sleepiness, depressive symptoms, OSA severity, and comorbidities and even though participants were enrolled in the study for a period of six months.

This is the first study demonstrating the favorable effect of incorporating a dietary/lifestyle intervention in combination with standard care towards objective PAP adherence in Greece. The findings suggest that providing intensive support with additional time allocated to the MDG may prove beneficial in enhancing PAP compliance, irrespective of weight loss. While previous studies with comparable follow-up times have investigated the impact of diet and lifestyle interventions alongside usual care on various OSA severity and cardiometabolic parameters, there has been no objective evaluation of PAP adherence [17,18,27,28,29]. Only self-reported PAP adherence was reported in a limited number of studies [17,18,28,29]. Compared to our study, Georgoulis et al. found a lower self-reported average PAP use of roughly 4 h/day [17]. Likewise, they observed a greater PAP compliance in the Mediterranean lifestyle group when compared to the Mediterranean diet alone and standard care group six months following the intervention [18]. Although Schiavo et al., in a similar study investigating the effect of a low-calorie ketogenic diet combined with PAP therapy on OSA and cardiometabolic parameters, acknowledged that they monitored adherence to PAP treatment, they did not provide relevant data [30]. Moreover, it is significant to observe that PAP adherence, even in the SCG, was substantially higher than in earlier studies [31,32]. The SCG’s adherence of 5.4 h per night is comparable to the one reported in a previous study from our group [21]. This can be attributed to the comprehensive, standardized approach of our PAP clinic. This approach includes personalized counseling and education on the proper use and maintenance of PAP, formal mask fitting, and frequent follow-ups to address residual symptoms, side effects, and the patients’ overall health status.

Despite recommendations [33] and studies indicating better clinical outcomes [34] with diet-induced weight loss as part of OSA treatment, interventions targeting weight loss through lifestyle changes are underutilized. Indeed, based on MeditDietScore we found medium adherence to the Mediterranean diet in our patients, suggesting that non-conventional care was not regarded as an essential component of overall patient care. Furthermore, another challenge is the lack of specific educational skills from health care professionals to support diet/lifestyle interventions or promote behavioral changes in these patients. Optimizing OSA treatment is crucial for clinicians, but many fail to recognize the importance of incorporating diet and lifestyle interventions into their treatment plans. Thus, our study has the potential to raise awareness regarding the importance of diet and lifestyle among OSA patients who are overweight or obese. Our results also demonstrated that the Mediterranean diet/lifestyle intervention contributed to improvements in BMI and BP measurements compared with standard care. These benefits were evident even after adjustment for confounders, suggesting that the Mediterranean lifestyle can lead to cardiovascular benefits beyond weight loss in these patients. These findings are noteworthy as they align with prior research indicating more weight loss in patients following diet/lifestyle intervention than standard care [16,17,18,27,30]. Although the weight loss achieved in our study was below the recommended 5–10% for managing obesity and achieving health benefits, even a slight reduction in weight was seen as beneficial for health, particularly among patients with OSA [17,35,36]. Another study we conducted involved randomly assigning 40 obese individuals with OSA undergoing PAP treatment to either a weight-loss Mediterranean diet or a weight-loss prudent diet for 6 months. The group on the Mediterranean diet showed more significant weight loss, but the results were not statistically significant due to a small number of participants [37]. In the MIMOSA RCT conducted recently, a dietary/lifestyle intervention also based on the Mediterranean pattern was combined with PAP treatment for OSA patients, resulting in a notable improvement in weight reduction, OSA severity, and related symptoms when compared to standard care alone [16,38].

At present, the prescription of PAP remains the first-line treatment for patients with moderate to severe OSA. However, effectiveness is dependent on the patient’s usage. Consequently, it is essential to take into account diet/lifestyle intervention as an added strategy in the treatment of OSA. These interventions provide a non-invasive approach that may also help to address PAP adherence issues. Combining diet and exercise has been found to be an effective intervention in mitigating OSA severity [34], therefore, it is probable that lower pressures will be required in the PAP machine [39], making it easier for patients to tolerate high PAP pressures. In support of this, evidence suggests that patients diagnosed with OSA frequently encounter distress and are incapable of tolerating heightened PAP pressures, thus abandoning PAP devices [40]. In addition, our intervention group received personalized and consistent guidance from a specialized dietitian, leading to better PAP adherence. Previously, in a 2-year study, our group found that intensive follow-up support was more effective in improving long-term PAP adherence than standard support [21]. It is plausible that the unequal intervention time spent between the SCG and the MDG in the current study may partially account for our results. The MDG received additional visits and hours of contact compared with the SCG, potentially influencing adherence outcomes. Therefore, the regular implementation of dietary and lifestyle interventions is a valuable strategy for treating OSA and must be consistently applied across all OSA patients.

The main strengths of the current study are the design (RCT) and implementation of a diet and lifestyle intervention readily adaptable to real-world clinical practice. On the other hand, the study has some limitations that deserve comments. Since it was a real-life implementation of a diet/lifestyle program, the results can only be generalized to patients who are motivated to participate in such a program and not to all OSA patients. In addition, the generalizability of our results is limited, due to the fact that the trial was conducted at a single center with a predominantly male patient population. Female under-representation is a common challenge in many studies, with women being referred less for OSA diagnosis and treatment compared to males [41]. Thus, considering the gender bias in the existing literature towards male subjects, it is essential that future studies encompass a more heterogeneous sample that includes both males and females with OSA.

Furthermore, this study was carried out during the second wave of the COVID-19 pandemic, which resulted in a small sample size. Additionally, a 6-month period was insufficient to determine long-term intervention effects and maintenance of benefits. Consequently, large-scale studies may require prolonged follow-up periods to assess the durability and sustainability of the observed improvements in PAP adherence, BMI, and blood pressure.

## 5. Conclusions

In conclusion, our results provide evidence that overweight/obese patients with moderate to severe OSA can benefit significantly in terms of PAP adherence, BP, and BMI control from behavioral interventions aiming at weight loss through the adoption of appropriate food and lifestyle practices. Therefore, it is essential to consider such type of intervention as an add-on approach to OSA management. However, additional evidence is needed from studies, including larger numbers of patients with longer-term follow-ups to explore the influence of diet/lifestyle interventions on OSA, and especially on the long-term sequelae.

## Figures and Tables

**Table 1:** Baseline anthropometric and clinical characteristics of the study population.

	All OSA Patients (*n* = 74)	OSA SCGroup (*n* = 37)	OSA MDGroup (*n* = 37)	*p* Value
**Demographics**				
Gender, males (%)	58 (78%)	36 (97%)	22 (60%)	<0.001
Age (years)	53 ± 11	53 ± 10	53 ± 13	0.79
Age ≥ 60 years	24 (32%)	10 (27%)	14 (38%)	0.32
BMI (kg/m^2^)	36 ± 8	35 ± 8	38 ± 9	0.16
BMI ≥ 30	57 (77%)	28 (76%)	29 (78%)	0.78
Neck circumference (cm)	42 ± 4	42 ± 3	43 ± 4	0.54
Waist circumference (cm)	120 ± 16	120 ± 17	121± 15	0.65
Hip circumference (cm)	120 ± 17	118 ± 16	123 ± 18	0.16
Waist/hip circumference ratio	1.0 ± 0.07	1.0 ± 0.06	0.99 ± 0.07	0.07
**Educational Level**				
Primary level or less	10 (13%)	7 (19%)	3 (8%)	
Secondary level	38 (52%)	18 (49%)	20 (54%)	
Tertiary level or higher	26 (35%)	12 (32%)	14 (38%)	0.75
**Smoking status**				
Never, *n* (%)	26 (36%)	11 (30%)	15 (40%)	
Currently smoking, *n* (%)	21 (28%)	10 (27%)	11 (30%)	
Former, *n* (%)	26 (36%)	16 (43%)	11 (30%)	0.36
Pack-years	15 (0, 39)	15 (0, 40)	13 (0, 39)	0.49
**Co-morbidities**				
Hypertension	36 (49%)	19 (51%)	17 (46%)	0.64
Coronary heart disease	5 (7%)	1 (3%)	4 (11%)	0.17
Atrial fibrillation	8 (11%)	2 (5%)	6 (16%)	0.13
Cardiovascular disease	20 (27%)	5 (14%)	15 (41%)	0.009
Diabetes type II	11 (15%)	5 (14%)	6 (16%)	0.74
COPD	16 (22%)	9 (24%)	7 (19%)	0.57
Bronchial asthma	10 (14%)	5 (14%)	5 (14%)	1.00
Hypothyroidism	11 (15%)	4 (11%)	7 (19%)	0.33
Dyslipidemia	29 (39%)	17 (46%)	12 (32%)	0.23
Depression	8 (11%)	2 (5%)	6 (16%)	0.13
**BP measurements**				
SBP (mmHg)	126 ± 14	127 ± 16	124 ± 13	0.37
DBP (mmHg)	78 ± 10	78 ± 11	77 ± 9	0.60

Data are presented as mean values ± SD or median (25th–75th percentile), unless otherwise indicated. BMI: body mass index, COPD: chronic obstructive pulmonary disease, BP: blood pressure, SBP: systolic blood pressure, DBP: diastolic blood pressure.

**Table 2:** Baseline polysomnography characteristics of the study population.

	All OSA Patients (*n* = 74)	OSA SCGroup (*n* = 37)	OSA MDGroup (*n* = 37)	*p* Value
Total recording time (min)	406 ± 40	404 ± 47	410 ± 31	0.53
Total sleep time (min)	276 ± 52	274 ± 52	280 ± 52	0.65
Sleep efficiency, %	68 ± 12	68 ± 12	68 ± 12	0.95
Wake after sleep onset time (min)	91 ± 36	95 ± 40	89 ± 33	0.53
Sleep latency	35 (24, 62)	33 (25, 57)	35 (24, 67)	0.75
REM latency	232 ± 79	231 ± 76	232 ± 83	0.96
NREM (%)	91 ± 3	91 ± 3	91 ± 3	0.75
REM (%)	9 ± 3	9 ± 3	9 ± 3	0.75
AHI	47 ± 24	46 ± 22	49 ± 27	0.60
REM AHI	54 ± 26	54 ± 26	55 ± 27	0.89
Arousal index	45 ± 17	46 ± 13	46 ± 20	0.95
Oxygen desaturation index	44 (29, 65)	46 (32, 66)	43 (29, 82)	0.83
Mean SaO_2_	92 ± 3	92 ± 2	91 ± 3	0.39
Lowest SaO_2_	80 (71, 83)	80 (72, 83)	79 (69, 83)	0.58
TST90 (min)	77 (34, 137)	74 (29, 114)	81 (36, 166)	0.53
**Severity of OSA (%)**				
15 ≤ AHI < 30	18 (24%)	8 (22%)	10 (27%)	
AHI ≥ 30	56 (76%)	29 (78%)	27 (73%)	0.59

OSA: obstructive sleep apnea, AHI: apnea-hypopnea index, TST90: sleep time with oxygen saturation below 90%.

**Table 3:** Nocturnal and diurnal symptoms of the study population.

	All OSA Patients (*n* = 74)	OSA SCGroup (*n* = 37)	OSA MDGroup (*n* = 37)	*p* Value
**Nocturnal Symptoms**				
Snoring	74 (100%)	37 (100%)	37 (100%)	1
Witnessed apneas	73 (99%)	36 (97%)	37 (100%)	0.31
Frequent awakenings	39 (53%)	15 (41%)	24 (65%)	0.04
Nocturia	67 (91%)	33 (89%)	34 (92%)	0.69
**Diurnal symptoms**				
ESS score	11 ± 5	12 ± 5	11 ± 5	0.43
ESS > 10	43 (58%)	23 (62%)	20 (54%)	0.48
Morning headache	51 (69%)	25 (68%)	26 (70%)	0.80
Driving problems	1 (1%)	0 (0%)	1 (3%)	0.31
BDI score	8 (3, 14)	5 (3, 18)	9 (5, 12)	0.25
BDI ≥ 10	25 (39%)	10 (31%)	15 (47%)	0.20

ESS: Epworth Sleepiness Scale, BDI: Beck Depression Inventory.

**Table 4:** Factors associated with optimal adherence to treatment at the end of the follow-up period in all patients.

Variable	B	S.E.	*p*-Value	OR (95% CI)
Females vs. males	−0.70	1.024	0.945	0.932 (0.125–26.036)
Age (years)	0.006	0.036	0.870	1.006 (0.938–1.079)
Baseline BMI (kg/m^2^)	0.094	0.057	0.101	1.099 (0.982–1.229)
Currently smoking vs. never/formerly smoking	−1.589	0.811	0.050	0.204 (0.042–1.001)
Baseline ESS score > 10	1.047	0.694	0.131	2.850 (0.732–11.098)
Baseline BDI score ≥ 10	−1.649	0.816	0.043	0.192 (0.039–0.951)
Arterial hypertension	1.037	0.778	0.183	2.821 (0.614–12.968)
Cardiovascular disease	−1.103	0.813	0.175	0.332 (0.067–1.634)
Type 2 diabetes	0.372	0.937	0.691	1.451 (0.231–9.109)
COPD	−0.655	0.917	0.475	0.519 (0.086–3.133)
MDG vs. SCG	1.697	0.797	0.033	5.458 (1.144–26.036)
Severe vs. moderate OSA	−0.625	0.863	0.469	0.535 (0.099–2.907)

## Data Availability

Data are available upon request.
